# ﻿Morphological and phylogenetic analyses reveal two new species of *Camporesiomyces* (Tubeufiaceae, Tubeufiales) from terrestrial habitats in China

**DOI:** 10.3897/mycokeys.124.168385

**Published:** 2025-10-23

**Authors:** Song Bai, Fang Wang, Su-Ran Wan, Xiao-Kang Lv, Li-Jun Chen, Rong Wu, Jian Ma

**Affiliations:** 1 Guizhou Industry Polytechnic College, Guiyang 550008, China Guizhou Industry Polytechnic College Guiyang China; 2 School of Food and Pharmaceutical Engineering, Guizhou Institute of Technology, Guiyang 550003, China School of Food and Pharmaceutical Engineering, Guizhou Institute of Technology Guiyang China

**Keywords:** Asexual morph, Dothideomycetes, phylogeny, saprobic fungi, taxonomy, two new species

## Abstract

During a survey of saprobic fungi in karst landscapes of Guizhou Province, China, fresh specimens were collected from decaying wood in terrestrial habitats. Phylogenetic analyses of a combined dataset (ITS, LSU, *tef*1-α and *rpb*2) along with morphological characteristics support the introduction of two novel species, *Camporesiomyces qizhifengensis* and *C. yusheensis*. Detailed descriptions, illustrations and phylogenetic evidence are provided to substantiate their taxonomic placement. Additionally, a checklist of currently accepted *Camporesiomyces* species supported by molecular data is included. This is the first report of *Camporesiomyces* in Guizhou, adding to the regional biodiversity of karst habitats and highlighting the ecological importance of these unique environments. The findings underscore the need for continued exploration of fungal diversity in underexplored regions.

## ﻿Introduction

Karst landscapes are primarily shaped by the dissolution of soluble rocks, such as limestone, dolomite and gypsum, through the action of groundwater or surface water. These landscapes span the provinces of Chongqing, Guangxi, Guizhou and Yunnan in China (Yang et al. 2023; Ma et al. 2024a, 2024b; Wang et al. 2024a, 2024b). Amongst them, Guizhou Province, with 62% of its land area (109,100 km^2^) covered by karst landscapes, is known for its rich biodiversity (Liu et al. 2018, 2020; Yang et al. 2023). Previous studies have explored the diversity, taxonomy and phylogeny of fungi in karst regions, primarily focusing on the development of freshwater fungal communities, with limited reports on the discovery of terrestrial fungi (Ma et al. 2022, 2023a, 2023b, 2023c, 2024a, 2024b; Calabon et al. 2022, 2023; Xiao et al. 2023; Xu et al. 2025).

*Camporesiomyces* was established by Hyde et al. (2020) to accommodate the type species, *C. mali*, along with two new combinations (*C. patagonicus* and *C. vaccinii*), based on morphological comparisons and phylogenetic analyses of combined ITS, LSU, *tef*1-α and *rpb*2 sequences. Previously, *Camporesiomyces patagonicus* and *C. vaccinii* were classified as *Acanthostigma patagonicum* and *Helicoma vaccinii*, respectively (Carris 1989; Sánchez et al. 2012; Hyde et al. 2020). Amongst them, *Camporesiomyces vaccinii* (≡ *Helicoma vaccinii*) was initially described by Carris (1989), with subsequent records from Peru and China in terrestrial habitats by Matsushima (1993) and Zhao et al. (2007), respectively. Tsui and Berbee (2006) were the first to provide ITS and LSU sequence data for *Helicoma vaccinii* (CBS 216.90), though without a morphological description. Later, CBS 216.90 was initially identified as *Helicosporium vaccinii* (Boonmee et al. 2014; Brahmanage et al. 2017; Chaiwan et al. 2017; Lu et al. 2017a). Following morphological comparisons to other asexual helicosporous species, Lu et al. (2018b) re-identified CBS 216.90 as *Helicoma vaccinii*, now *C. vaccinii*. Recently, Han et al. (2025) introduced three new species of *Camporesiomyces*: *C. bhatii*, *C. coffeae* and *C. puerensis*, which were isolated from dead branches of *Coffea arabica* and *Coffea liverica* in terrestrial habitats in Yunnan Province, China. Currently, six species are accepted within the genus *Camporesiomyces* (Han et al. 2025).

Morphologically, species of *Camporesiomyces* exhibit both asexual and sexual morphs, with two distinct asexual morphs (Carris 1989; Matsushima 1993; Zhao et al. 2007; Hyde et al. 2020; Han et al. 2025). The sexual morph is characterised by multi-loculate, black, subglobose to conical ascomata, bitunicate, fissitunicate, cylindrical asci and narrowly fusiform, hyaline, multi-septate ascospores (Hyde et al. 2020). The asexual morphs are characterised as follows: type 1) macronematous, mononematous, branches or unbranched, septate conidiophores, monoblastic or polyblastic, denticulate conidiogenous cells, and helicoid, solitary, guttulate, septate, hyaline conidia (Carris 1989; Matsushima 1993; Zhao et al. 2007; Hyde et al. 2020); and type 2) macronematous, mononematous, erect, solitary, unbranched septate, conidiophores, polyblastic, integrated, denticulate, terminal conidiogenous cells and acrogenous, solitary, cylindrical, obclavate or fusiform, septate, guttulate conidia (Han et al. 2025).

In this study, four asexual isolates, representing two distinct taxa, were obtained from decaying wood in terrestrial habitats in Qizhifeng Forest Park and Yushe National Forest Park, Guizhou Province. Based on morphological characteristics, illustrations and phylogenetic analyses using Maximum Likelihood and Bayesian Inference of combined ITS, LSU, *tef*1-α and *rpb*2 sequence data, two novel species are introduced, namely, *Camporesiomyces qizhifengensis* and *C. yusheensis*.

## ﻿Materials and methods

### ﻿Sample collection, examination and isolation

Decaying wood samples were collected in November 2024 from Liupanshui City, Guizhou Province, south-western China. Fresh samples were transported to the laboratory in plastic bags with the collection details, including localities, habitats and dates (Rathnayaka et al. 2024). The microscopic features were examined and photographed using a stereomicroscope (SMZ-168, Nikon, Japan) and an ECLIPSE Ni compound microscope (Nikon, Tokyo, Japan) with a Canon 90D digital camera. Measurements were made using Tarosoft (R) Image Frame Work software. Photo-plates were made using Adobe Photoshop CC 2019 (Adobe Systems, USA).

Single conidium isolates were done on PDA (potato dextrose agar) plates following the methods described by Chomnunti et al. (2014) and Senanayake et al. (2020) and the germinated conidia were aseptically transferred to fresh PDA plates. Morphological characteristics of fungal mycelium on PDA, including colony colour, hyphal shape and growth dimensions, were recorded. Dried fungal specimens were deposited in the Herbarium of Kunming Institute of Botany, Chinese Academy of Sciences (Herb. HKAS) in Kunming, China and Herbarium of Guizhou Academy of Agriculture Sciences (Herb. GZAAS), Guiyang, China. Pure cultures were preserved in the Guizhou Culture Collection, China (GZCC), Guiyang, China. The MycoBank numbers were obtained as described in https://www.mycobank.org/.

### ﻿DNA extraction, PCR amplification and sequencing

Fresh fungal mycelium was scraped from colonies grown on PDA plates and transferred to a 1.5 ml microcentrifuge tube using a sterilised lancet for genomic DNA extraction. Genomic DNA was extracted using the Biospin Fungus Genomic DNA Extraction Kit (BioFlux, China). The following primer pairs were used to amplify specific gene regions: ITS5/ITS4 for the internal transcribed spacer (ITS; White et al. 1990), LR0R/LR5 for the large ribosomal subunit (LSU; Vilgalys and Hester 1990), EF1-983F/EF1-2218R for translation elongation factor 1-α (*tef*1-α; Rehner and Buckley 2005) and fRPB2-5F/fRPB2-7cR for RNA polymerase II second largest subunit (*rpb*2; Liu et al. 1999). DNA preparation was conducted in a 25 μl mixture, which included 1 μl of DNA, 1 μl of each forward and reverse primer and 22 μl of 1.1× T3 Super PCR Mix (Qingke Biotech, Chongqing, China). Polymerase chain reaction (PCR) was performed using the cycling conditions described by Ma et al. (2024a). The PCR products were purified and sequenced with the same primers at Beijing Tsingke Biotechnology Co., Ltd.

### ﻿Phylogenetic analyses

The newly-obtained sequences were quality-checked and assembled using BioEdit v.7.0.5.3 (Hall 1999) and SeqMan v.7.0.0 (DNASTAR, Madison, WI, USA; Swindell and Plasterer 1997), respectively. The sequences used in this study were retrieved from GenBank (Table 1; https://www.ncbi.nlm.nih.gov/). Sequence matrices for each gene were aligned using MAFFT v.7.473 (https://mafft.cbrc.jp/alignment/server/; Katoh et al. (2019)). Each gene dataset was trimmed using trimAl v.1.2rev59 software (Capella-Gutiérrez et al. 2009). A concatenated sequence dataset was generated using SequenceMatrix-Windows-1.7.8 software (Vaidya et al. 2011).

**Table 1.** Taxa used in this study and their GenBank accession numbers.

Taxon	Strain	GenBank Accession Numbers				References
		ITS	LSU	tef1-α	rpb2	
*Acanthohelicospora aurea*	GZCC 16-0060	KY321323	KY321326	KY792600	MF589911	Lu et al. (2017a)
*Acanthostigma chiangmaiensis*	MFLUCC 10-0125^T^	JN865209	JN865197	KF301560	N/A	Boonmee et al. (2014)
*Acanthostigma perpusillum*	UAMH 7237	AY916492	AY856892	N/A	N/A	Tsui et al. (2006)
*Berkleasmium aquaticum*	MFLUCC 17-0049^T^	KY790444	KY790432	KY792608	MF535268	Lu et al. (2017b)
*Berkleasmium fusiforme*	MFLUCC 17-1978^T^	MH558693	MH558820	MH550884	MH551007	Lu et al. (2018b)
*Boerlagiomyces macrospora*	MFLUCC 12-0388	KU144927	KU764712	KU872750	N/A	Doilom et al. (2017)
*Botryosphaeria agaves*	MFLUCC 10-0051	JX646790	JX646807	N/A	N/A	Liu et al. (2012)
*Botryosphaeria dothidea*	CBS 115476	KF766151	DQ678051	DQ767637	DQ677944	Slippers et al. (2013)
*Camporesiomyces bhatii*	GMBCC 1120^T^	PQ763360	PQ842543	PV388894	PV388888	Han et al. (2025)
*Camporesiomyces bhatii*	GMBCC 1125	PQ763361	PQ842544	PV388895	PV388889	Han et al. (2025)
*Camporesiomyces coffeae*	GMBCC 1130^T^	PQ763358	PQ842545	PV388896	PV388890	Han et al. (2025)
*Camporesiomyces coffeae*	GMBCC 1131	PQ763359	PQ842546	PV388897	PV388891	Han et al. (2025)
*Camporesiomyces mali*	KUMCC 19-0216^T^	NR_169709	NG_075312	MN794018	N/A	Hyde et al. (2020)
*Camporesiomyces patagonicus*	BBB MVB 573	JN127358	JN127359	N/A	N/A	Hyde et al. (2020)
*Camporesiomyces puerensis*	GMBCC 1113^T^	PQ763356	PQ842541	PV388892	PV388886	Han et al. (2025)
*Camporesiomyces puerensis*	GMBCC 1114	PQ763357	PQ842542	PV388893	PV388887	Han et al. (2025)
***Camporesiomyces qizhifengensis***	**GZCC 25-0638^T^**	**PX111185**	**PX111192**	**PX102609**	**N/A**	**In this study**
***Camporesiomyces qizhifengensis***	**GZCC 25-0639**	**PX111186**	**PX111193**	**PX102610**	**N/A**	**In this study**
*Camporesiomyces vaccinii*	CBS 216.90	MH862204	MH873889	N/A	N/A	Hyde et al. (2020)
***Camporesiomyces yusheensis***	**GZCC 25-0636^T^**	**PX111183**	**PX111190**	**PX102607**	**PX102601**	**In this study**
***Camporesiomyces yusheensis***	**GZCC 25-0637**	**PX111184**	**PX111191**	**PX102608**	**PX102602**	**In this study**
*Chlamydotubeufia cylindrica*	MFLUCC 16-1130^T^	MH558702	MH558830	MH550893	MH551018	Lu et al. (2018b)
*Chlamydotubeufia huaikangplaensis*	MFLUCC 10-0926^T^	JN865210	JN865198	N/A	N/A	Boonmee et al. (2011)
*Dematiohelicomyces helicosporus*	MFLUCC 16-0213^T^	KX454169	KX454170	KY117035	MF535258	Hyde et al. (2016)
*Dematiohelicosporum guttulatum*	MFLUCC 17-2011^T^	MH558705	MH558833	MH550896	MH551021	Lu et al. (2018b)
*Dematiotubeufia chiangraiensis*	MFLUCC 10-0115^T^	JN865200	JN865188	KF301551	N/A	Boonmee et al. (2011)
*Helicangiospora lignicola*	MFLUCC 11-0378^T^	KF301523	KF301531	KF301552	N/A	Boonmee et al. (2014)
*Helicoarctatus aquaticus*	MFLUCC 17-1996^T^	MH558707	MH558835	MH550898	MH551024	Lu et al. (2018b)
*Helicohyalinum aquaticum*	MFLUCC 16-1131^T^	KY873625	KY873620	KY873284	MF535257	Lu et al. (2018b)
*Helicohyalinum infundibulum*	MFLUCC 16-1133^T^	MH558712	MH558840	MH550903	MH551029	Lu et al. (2018b)
*Helicoma guttulatum*	MFLUCC 16-0022^T^	KX454171	KX454172	MF535254	MH551032	Hyde et al. (2016)
*Helicoma hongkongense*	MFLUCC 17-2005	MH558716	MH558843	MH550907	MH551033	Lu et al. (2018b)
*Helicosporium acropleurogenum*	CGMCC 3.25563^T^	PP626574	PP639430	PP596333	PP596460	Ma et al. (2024b)
*Helicosporium aquaticum*	MFLUCC 17-2008^T^	MH558733	MH558859	MH550924	MH551049	Lu et al. (2018b)
*Helicosporium brunneisporum*	CGMCC 3.25542^T^	PP626577	PP639433	PP596336	PP596463	Ma et al. (2024b)
*Helicosporium changjiangense*	GZCC 22-2113^T^	PP626578	PP639434	PP596337	PP596464	Ma et al. (2024b)
*Helicosporium flavisporum*	MFLUCC 17-2020^T^	MH558734	MH558860	MH550925	MH551050	Lu et al. (2018b)
*Helicosporium ramosiphorum*	CGMCC 3.25541^T^	PP626576	PP639432	PP596335	PP596462	Ma et al. (2024b)
*Helicosporium rubrum*	MFLUCC 24-0090^T^	PQ098477	PQ098514	PQ490681	PQ490675	Peng et al. (2025)
*Helicosporium setiferum*	MFLUCC 17-1994^T^	MH558735	MH558861	MH550926	MH551051	Tsui et al. (2006)
*Helicosporium sexuale*	MFLUCC 16-1244^T^	MZ538503	MZ538537	MZ567082	MZ567111	Boonmee et al. (2011)
*Helicotubeufia hydei*	MFLUCC 17-1980^T^	MH290021	MH290026	MH290031	MH290036	Liu et al. (2018)
*Helicotubeufia jonesii*	MFLUCC 17-0043^T^	MH290020	MH290025	MH290030	MH290035	Liu et al. (2018)
*Muripulchra aquatica*	MFLUCC 15-0249^T^	KY320532	KY320549	N/A	N/A	Luo et al. (2017)
*Neoacanthostigma fusiforme*	MFLUCC 11-0510^T^	KF301529	KF301537	N/A	N/A	Boonmee et al. (2014)
*Neochlamydotubeufia fusiformis*	MFLUCC 16-0016^T^	MH558740	MH558865	MH550931	MH551059	Lu et al. (2018b)
*Neohelicomyces acropleurogenus*	CGMCC 3.25549^T^	PP626594	PP639450	PP596351	PP596478	Ma et al. (2024b)
*Neohelicomyces aquaticus*	MFLUCC 16-0993^T^	KY320528	KY320545	KY320561	MH551066	Luo et al. (2017)
*Neohelicosporium acrogenisporum*	MFLUCC 17-2019^T^	MH558746	MH558871	MH550937	MH551069	Lu et al. (2018b)
*Neohelicosporium aquaticum*	MFLUCC 17-1519^T^	MF467916	MF467929	MF535242	MF535272	Lu et al. (2018a)
*Neomanoharachariella xizangensis*	KUNCC 23-15799^T^	OR803724	OR803722	OR813978	OR813975	Yang et al. (2024)
*Parahelicomyces quercus*	MFUCC 17-0895^T^	MK347720	MK347934	MK360077	MK434906	Hsieh et al. (2021)
*Parahelicomyces talbotii*	MFLUCC 17-2021^T^	MH558765	MH558890	MH550957	MH551091	Hsieh et al. (2021)
*Tubeufia guttulata*	GZCC 23-0404^T^	OR030841	OR030834	OR046678	OR046684	Ma et al. (2023c)
*Tubeufia hainanensis*	GZCC 22-2015^T^	OR030842	OR030835	OR046679	OR046685	Ma et al. (2023c)
*Zaanenomyces moderatricis-academiae*	CPC 41273^T^	OK664723	OK663762	N/A	OK651167	Crous et al. (2023)
*Zaanenomyces versatilis*	CPC 41224^T^	OK664730	OK663769	N/A	N/A	Crous et al. (2023)

Note: “^T^” indicates ex-type strains. Newly-generated sequences are in bold. “N/A” indicates the unavailable data in GenBank.

Maximum Likelihood (ML) analysis was performed using IQ-TREE web server (http://iqtree.cibiv.univie.ac.at/) with the best-fit substitution model automatically selected based on the Bayesian Information Criterion (BIC) (Nguyen et al. 2015). Bayesian Inference (BI) analysis was conducted by using MrBayes on XSEDE (3.2.7a) via CIPRES (Stamatakis 2014). The aligned FASTA file was converted to a Nexus format file using AliView (Daniel et al. 2010). The optimal substitution model for each dataset was determined using MrModelTest v.2.3 (Nylander et al. 2008). The posterior probabilities (BYPP) were determined, based on Bayesian Markov Chain Monte Carlo (BMCMC) sampling (Huelsenbeck and Ronquist 2001). Four simultaneous Markov chains were run for 10,000,000 generations and trees were sampled every 1,000^th^ generation. The burn-in phase was set at 25% and the remaining trees were used for calculating posterior probabilities (BYPP).

Phylogenetic trees were visualised using FigTree v.1.4.4 and subsequently edited using Adobe Illustrator CC 2019 (v.23.1.0; Adobe Systems, USA).

## ﻿Phylogenetic results

**Figure 1.**
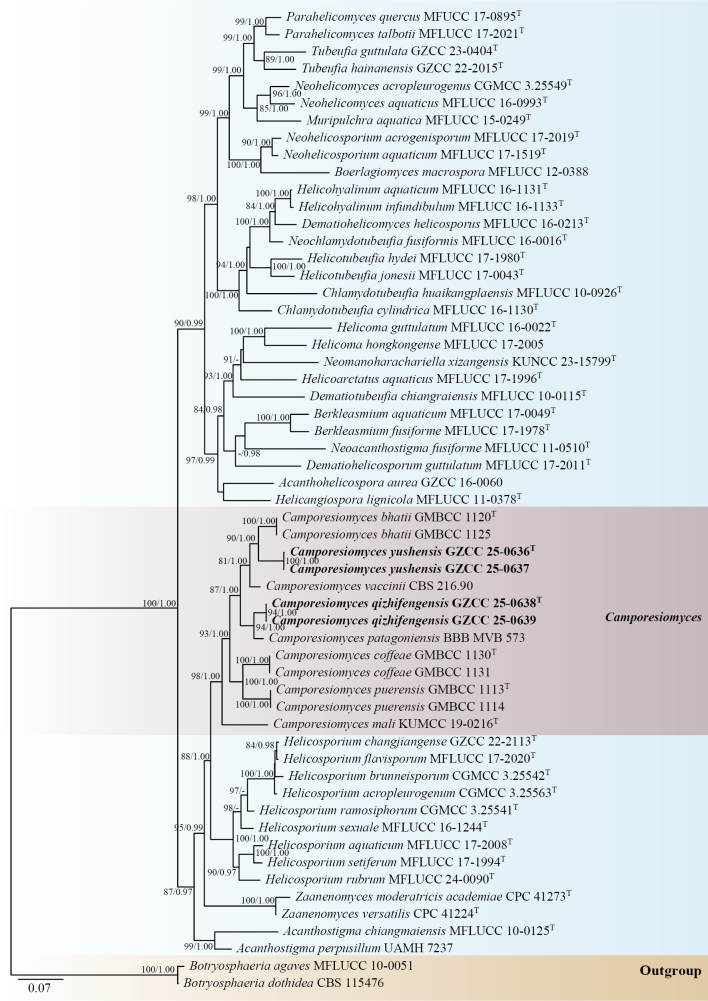
Phylogenetic tree generated from the ML analysis, based on the combined ITS, LSU, *tef*1-α and *rpb*2 sequence data. Bootstrap support values of ML equal to or greater than 75% and Bayesian posterior probabilities (PP) equal to or greater than 0.95 are given near the nodes as ML/BYPP, respectively. The Maximum Likelihood (ML) and Bayesian Inference (BI) analyses yielded similar tree topologies. Hyphen (“-”) indicates a value lower than 75% for ML and a posterior probability lower than 0.95 for BI. *Botryosphaeria agaves* (MFLUCC 10-0051) and *B. dothidea* (CBS 115476) were selected as outgroups (Lu et al. 2018b). Ex-type strains are denoted with “^T^” and newly-obtained isolates are in bold black fonts.

The phylogenetic positions of the four novel strains were determined through multi-locus phylogenetic analysis. The concatenated sequence matrix comprised 3,401 characters (ITS: 1–583, LSU: 584–1,444, *tef*1-α: 1,445–2,356, and *rpb*2: 2,357–3,401) across 57 taxa. Fig. 1 shows the best-scoring Maximum Likelihood (ML) tree with a final log-likelihood value of -33524.456.

Based on concatenated phylogenetic analysis of ITS, LSU, *tef*1-α and *rpb*2 loci (Fig. 1), our isolates represent two distinct species of *Camporesiomyces*, forming well-supported clades separate from known taxa. Isolates GZCC 25-0638 and GZCC 25-0639 formed a well-supported clade (ML = 94%, BYPP = 1.00) sister to *C. patagonicus* (BBB MVB 573). Additionally, isolates GZCC 25-0636 and GZCC 25-0637 formed a distinct lineage with *C. bhatii* (GMBCC 1120 and GMBCC 1125) with 90% ML and 1.00 BYPP support.

### ﻿Taxonomy

#### ﻿ Camporesiomyces qizhifengensis

Taxon classificationFungiTubeufialesTubeufiaceae

Song Bai, Rong Wu & Jian Ma sp. nov.

349A469B-9266-549C-956C-CCC7E300CD0B

904188

Fig. 2.

##### Etymology.

‘‘*qizhifengensis*” refers to the ‘‘Qizhifeng Forest Park” where the holotype was collected.

##### Holotype.

HKAS 128896.

##### Description.

***Saprobic*** on decaying wood in a terrestrial habitat. ***Sexual morph*** Not seen. ***Asexual morph*** hyphomycetous. ***Colonies*** on natural substratum superficial, effuse, scattered or aggregated, hairy, yellow. ***Mycelium*** partly superficial, partly immersed, composed of branched, septate, guttulate, smooth-walled, hyaline to brown hyphae. ***Conidiophores*** 65–122 × 4–6 μm (x̄ = 88 × 5.2 μm, n = 25), macronematous, mononematous, erect, solitary, smooth or occasionally verruculose, cylindrical, dark brown, paler towards apex, slightly flexuous, unbranched, 4–11-septate, sometimes slightly constricted at septa. ***Conidiogenous cells*** 10–18 × 2.8–6 μm (x̄ = 14.5 × 3.7 μm, n = 25), polyblastic, integrated, terminal, determinate, cylindrical, slightly tapering, conspicuously denticulate at conidial secession, subhyaline to pale brown. ***Conidia*** 11–16.2 × 3.9–5.2 μm (x̄ = 13.3 × 4.6 μm, n = 30), acrogenous, solitary, cylindrical, obclavate or fusiform, 0–4-septate, guttulate, subhyaline to yellowish-brown, slightly constricted at septa.

**Figure 2.**
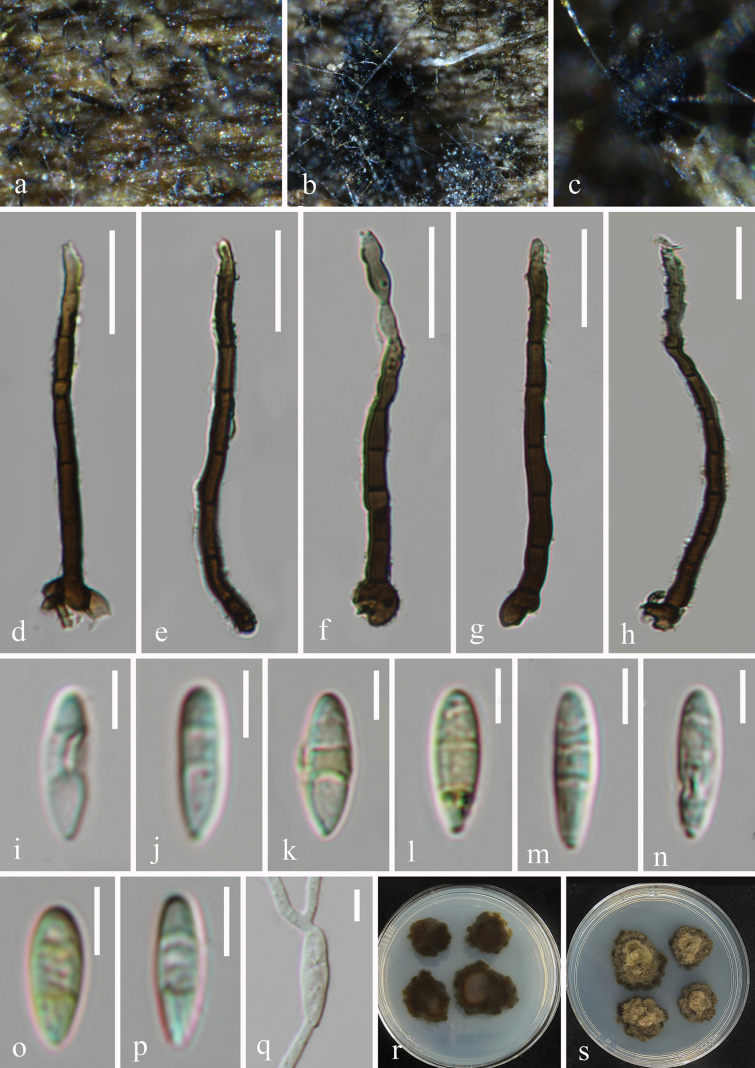
*Camporesiomyces qizhifengensis* (HKAS 128896, holotype). a–c. Colonies on the host surface; d–h. Conidiophores and conidiogenous cells; i–p. Conidia; q. Germinated conidium; r, s. Colonies on PDA from below and above after 39 days of incubation at room temperature. Scale bars: 20 μm (d–h); 5 μm (i–q).

##### Culture characteristics.

Conidia germinating on PDA within 18 hours, producing germ tubes from apices. Colony on PDA reaching 3 cm diam. after 39 days at room temperature (approximately 25 °C), circular or irregular, umbonate, with undulate margin, brown to dark brown, reverse pale brown to brown.

##### Material examined.

China • Guizhou Province, Liupanshui City, Dashan Town, Qizhifeng Forest Park, on decaying wood in a terrestrial habitat, 27 November 2024, Xia Tang, LQ05 (HKAS 128896, holotype; GZAAS 25–0669, isotype), ex-type living culture GZCC 25–0638; • *Ibid*., LQ09 (GZAAS 25–0666, paratype), living culture GZCC 25–0639.

##### Notes.

*Camporesiomyces qizhifengensis* (HKAS 128896) exhibits morphological similarities to *C. coffeae*, particularly in conidiophore and conidial morphology (Han et al. 2025). However, this species differs from *C. coffeae* in having longer conidiophores (up to 122 μm vs. 43–97 μm) and shorter conidia (11–16.2 μm vs. 20–50 μm) (Han et al. 2025). Our multi-gene phylogenetic analysis strongly supports that our isolates (GZCC 25–0638 and GZCC 25–0639) form a sister clade to *C. patagonicus*, with 94% ML and 1.00 BYPP support (Fig. 1). Additionally, base pair comparison between *C. qizhifengensis* and *C. patagonicus* reveals 14/322 bp differences in ITS (4.3%, including six gaps) and 6/613 bp differences in LSU (0.9%, without gaps), further supporting their distinction as separate species. The sexual state of *C. patagonicus* is the only known form of this species (Sánchez et al. 2012; Hyde et al. 2020; Ma et al. 2024b). Therefore, based on both the multi-gene phylogenetic analysis and morphological differences, we introduce *Camporesiomyces qizhifengensis* as a new species.

#### ﻿ Camporesiomyces yusheensis

Taxon classificationFungiTubeufialesTubeufiaceae

Song Bai, Rong Wu & Jian Ma sp. nov.

E12F46D4-DC0C-5537-88E7-50CE4188292C

904189

Fig. 3.

##### Etymology.

‘‘*yusheensis*” refers to the ‘‘Yushe National Forest Park” where the holotype was collected.

##### Holotype.

HKAS 128898.

##### Description.

***Saprobic*** on decaying wood in a terrestrial habitat. ***Sexual morph*** Not seen. ***Asexual morph*** hyphomycetous. ***Colonies*** on natural substratum superficial, effuse, scattered or aggregated, hairy, yellow at apex. ***Mycelium*** partly superficial, partly immersed, composed of branched, septate, guttulate, smooth-walled, hyaline to brown hyphae. ***Conidiophores*** 51–97 × 3.7–5.8 μm (x̄ = 69 × 4.6 μm, n = 30), macronematous, mononematous, erect, solitary, smooth or occasionally verruculose, cylindrical, dark brown, paler towards apex, slightly flexuous, unbranched, 4–12-septate, sometimes slightly constricted at septa. ***Conidiogenous cells*** 9.6–17.8 × 3.3–4.1 μm (x̄ = 13.4 × 3.6 μm, n = 25), polyblastic, integrated, terminal, determinate, cylindrical, slightly tapering, conspicuously denticulate at conidial secession, subhyaline to pale brown. ***Conidia*** 15.4–23 × 4.5–6.8 μm (x̄ = 19.3 × 5.3 μm, n = 35), acrogenous, solitary, cylindrical, obclavate or fusiform, 0–4-septate, mostly 3–4-septate, slightly flexuous, guttulate, brown to yellowish-brown.

**Figure 3.**
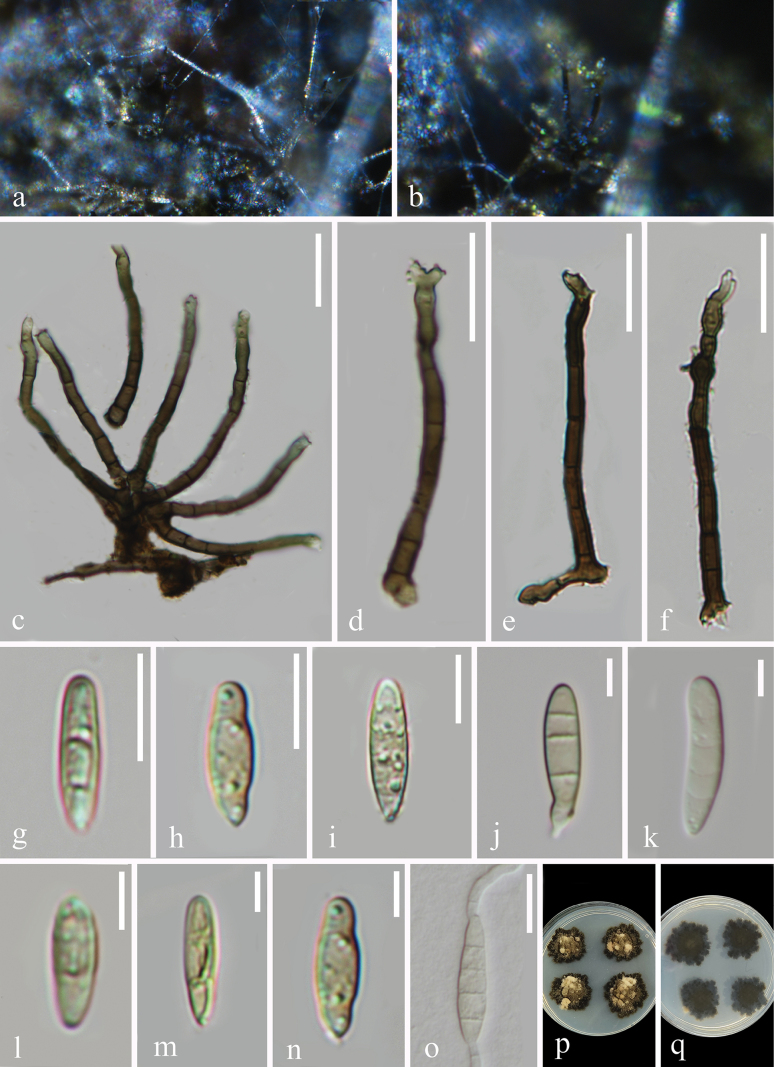
*Camporesiomyces yusheensis* (HKAS 128898, holotype). a, b. Colonies on the host surface; c–f. Conidiophores and conidiogenous cells; g–n. Conidia; o. Germinated conidium; p, q. Colonies on PDA from above and below after 41 days of incubation at room temperature. Scale bars: 20 μm (c–f); 10 μm (g–j); 5 μm (k–o).

##### Culture characteristics.

Conidia germinating on PDA within 15 hours, producing germ tubes from apices. Colony on PDA reaching 3.2 cm diam. after 41 days at room temperature (approximately 25 °C), circular or irregular, umbonate, with undulate margin, pale brown to black, reverse black.

##### Material examined.

China • Guizhou Province, Liupanshui City, Shuicheng County, Yushe National Forest Park, on decaying wood in a terrestrial habitat, 27 November 2024, Xia Tang, LS53 (HKAS 128898 holotype; GZAAS 25–0667, isotype), ex-type living culture GZCC 25–0636; *Ibid*., LS63 (GZAAS 25–0668, paratype), living culture GZCC 25–0637.

##### Notes.

In the phylogenetic analyses (Fig. 1), *Camporesiomyces yusheensis* formed a sister clade to *C. bhatii* with 90% ML and 1.00 BYPP support. However, a comparison of nucleotides in the ITS, LSU, *tef*1-α and *rpb*2 sequence between *C. yusheensis* and *C. bhatii*, revealed nucleotide differences of 25/494 bp (5.1%, including five gaps), 4/855 bp (0.5%, without gaps), 61/910 bp (6.7%, without gaps) and 87/958 bp (9.1%, without gaps), respectively. Moreover, *C. yusheensis* differs from *C. bhatii* by its shorter conidiogenous cells (9.6–17.8 μm vs. up to 21.7 μm) and shorter conidia (15.4–23 μm vs. up to 30 μm) (Han et al. 2025). Therefore, based on ITS, LSU, *tef*1-α and *rpb*2 sequence data and morphological characteristics, we introduce *Camporesiomyces yusheensis* as a new species.

## ﻿Discussion

Prior to this study, six species were recognised within *Camporesiomyces* and, with the addition of our two newly-described species, the genus now comprises eight species (Carris 1989; Matsushima 1993; Tsui and Berbee 2006; Zhao et al. 2007; Sánchez et al. 2012; Lu et al. 2018b; Hyde et al. 2020; Han et al. 2025). All *Camporesiomyces* species are found in terrestrial habitats, with primary distributions in Guizhou, Jilin and Yunnan Provinces of China and additional records from Argentina, China, Peru and the USA. They occur as saprobes on *Coffea arabica*, *C. liberica*, *Malus halliana*, *Nothofagus alpina*, *palma* and *Vaccinium elliotii* and decaying wood in terrestrial habitats.

Based on DNA molecular evidence, some helicosporous genera within Tubeufiaceae exhibit a wide range of conidial morphologies in their asexual reproductive forms (Lu et al. 2018b; Ma et al. 2024b; Han et al. 2025). For example, *Tubeufia* is characterised by muriform, dorsiventrally curved, coiled, ovate or ellipsoid, globose to subglobose and ovoid to irregular conidia, while *Camporesiomyces* is characterised by cylindrical, obclavate or fusiform or helicoid conidia (Carris 1989; Matsushima 1993; Zhao et al. 2007; Lu et al. 2018b; Ma et al. 2024b; Hyde et al. 2020; Han et al. 2025). This morphological diversification may be explained by molecular stasis, whereby genetic sequences remain relatively conserved while species undergo morphological adaptations in response to environmental pressures.

Morphologically, *Camporesiomyces vaccinii* resembles species of *Helicoma*, particularly in conidiophore and conidial features (Lu et al. 2018b; Ma et al. 2024b). However, *C. vaccinii* can be distinguished from *Helicoma* species by its long basal cell, which tapers towards the narrow base of the conidia (Carris 1989; Matsushima 1993; Zhao et al. 2007; Hyde et al. 2020; Han et al. 2025). Additionally, *C. vaccinii* differs from other asexual species of *Camporesiomyces* by possessing helicoid conidia (Carris 1989; Matsushima 1993; Zhao et al. 2007; Hyde et al. 2020; Han et al. 2025).

## Supplementary Material

XML Treatment for Camporesiomyces qizhifengensis

XML Treatment for Camporesiomyces yusheensis

## ﻿Additional information

### Use of AI

No use of AI was reported.

### Author ORCIDs

Song Bai https://orcid.org/0000-0002-1972-2834

Fang Wang https://orcid.org/0000-0002-3341-6788

Su-Ran Wan https://orcid.org/0009-0000-1164-6921

Xiao-Kang Lv https://orcid.org/0009-0006-7254-8747

Li-Jun Chen https://orcid.org/0009-0004-8562-875X

Rong Wu https://orcid.org/0000-0002-4946-8806

Jian Ma https://orcid.org/0009-0008-1291-640X

### Data availability

All of the data that support the findings of this study are available in the main text.
